# Incarcerated Amyand hernia with simultaneous rupture of an adenocarcinoma in an inguinal hernia sac: a case report

**DOI:** 10.1186/s13256-015-0592-x

**Published:** 2015-05-28

**Authors:** Ioannis Karanikas, Argyrios Ioannidis, Petros Siaperas, Georgios Efstathiou, Ioannis Drikos, Nicolaos Economou

**Affiliations:** Department of Surgery, Sismanoglion General Hospital, Sismanoglou 1, P.O. BOX 15126, Athens, Greece

**Keywords:** Amyand’s hernia, Colon cancer, Inguinal hernia, Right hemicolectomy

## Abstract

**Introduction:**

An Amyand’s hernia is a rare occurrence of an inguinal hernia, with an estimated prevalence of 1%. The major complications of an Amyand’s hernia include necrotizing fasciitis of the anterior abdominal wall and secondary intestinal perforation. Though the incidence of this type of hernia is low, the appendix may easily become initially incarcerated, possibly leading to strangulation and perforation.

**Case presentation:**

A 92-year-old female patient presented to our emergency department with clinical signs of an incarcerated right inguinal hernia, accompanied by fever. A clinical examination revealed localized abdominal pain, reflecting to the right side of her groin. Laboratory tests showed leukocytosis (13,200/μL), while an abdominal X-ray showed colon distension with evidence of intestinal obstruction. Ultrasonography was performed and confirmed the presence of an inflamed tubular structure inside her right inguinal canal. Our patient underwent emergency surgery. We started with a right inguinal incision, which revealed an incarcerated right inguinal hernia, containing her ruptured appendix and showing macroscopic evidence of malignancy. A specimen biopsy was immediately performed and the results showed a ruptured cecal adenocarcinoma. The incision was slightly extended upwards, and a right hemicolectomy performed.

**Conclusions:**

Diagnosis of an Amyand’s hernia occurs primarily as an incidental finding during surgery and the optimal therapeutic approach must be considered individually for each case. Owing to the rarity of Amyand’s hernia and the wide variance of its clinical characteristics, every case provides useful information toward the treatment of this type of hernia.

## Introduction

An Amyand’s hernia is an extremely rare condition in which the hernia sac contains the appendix. This rare surgical problem is estimated to occur in about 1% of adult patients who undergo surgery for an incarcerated inguinal hernia and has symptoms similar to those of acute appendicitis. Amyand’s hernias can be classified into four subtypes: (I) Amyand’s hernia with a normal-appearing appendix in the inguinal sac; (II) Amyand’s hernia with an inflamed appendix; (III) Amyand’s hernia with a perforation of the vermiform appendix; and (IV) complicating intra-abdominal pathology.

We describe a rare case of acute appendicitis with the simultaneous rupture of a cecal carcinoma in a patient with an Amyand’s hernia. Coexistence of a malignancy of the appendix with an Amyand’s hernia is an unusual condition because neoplasms of the appendix are very rare.

## Case presentation

A 92-year-old female patient presented to our emergency department with clinical evidence of an incarcerated right inguinal hernia, accompanied by fever and local skin inflammation.

**Figure 1:**
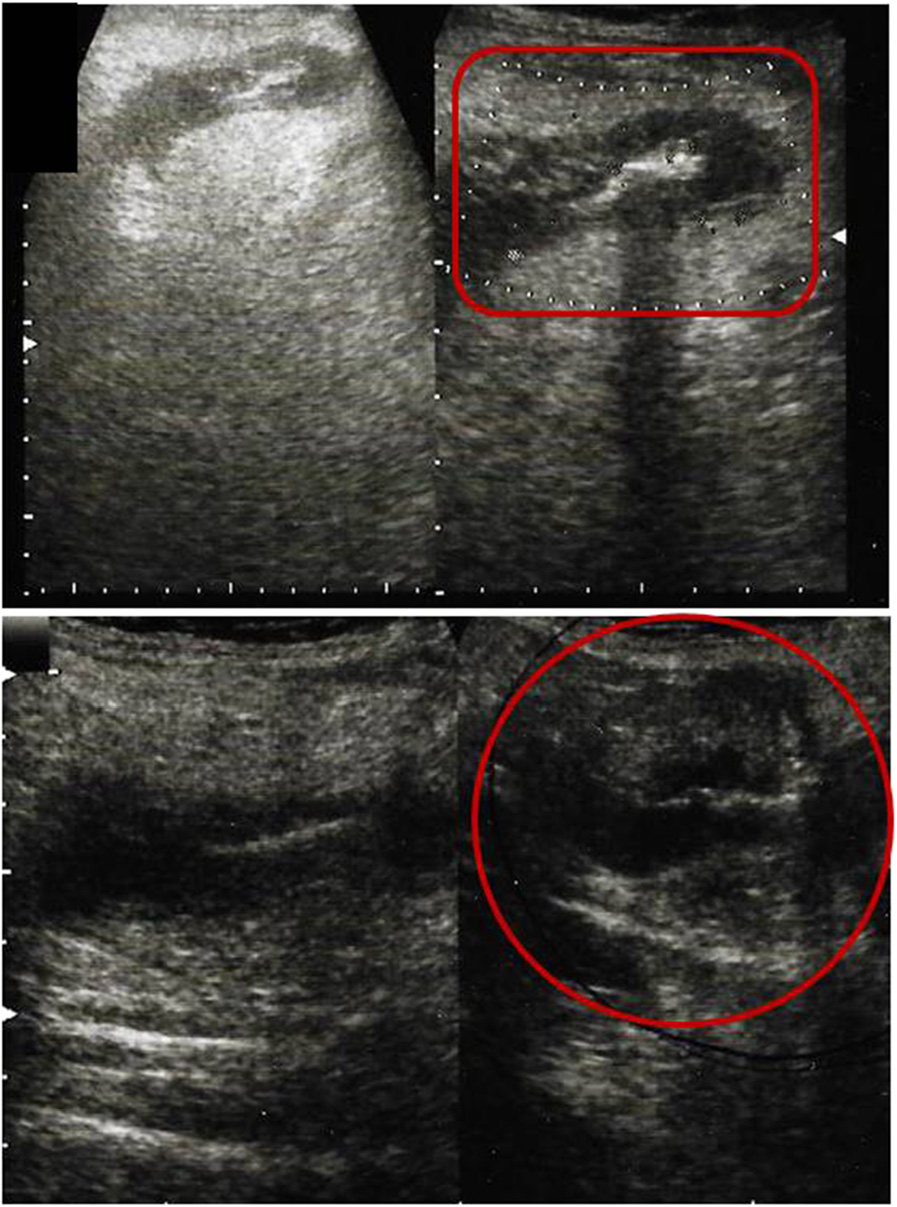
Ultrasonography revealed an inflamed tubular structure inside the right inguinal canal. This tubular structure looked similar to the appendix and was extending along the inguinal canal demonstrating the decision of emergency surgery treatment.

During a clinical examination, our patient reported abdominal tenderness, reflecting to the right side of her groin. Laboratory tests showed an increased number of white blood cells (13,200/μL, neutrophils 72%), while an abdominal X-ray revealed a distended colon and signs of intestinal obstruction. Groin ultrasonography showed an inflamed tubular structure in her right inguinal canal (Figure 1). This tubular structure looked similar to the appendix and was extending along her inguinal canal, entering her right iliac fossa, with no remarkable adjacent free fluid collection in her peritoneum.

**Figure 2:**
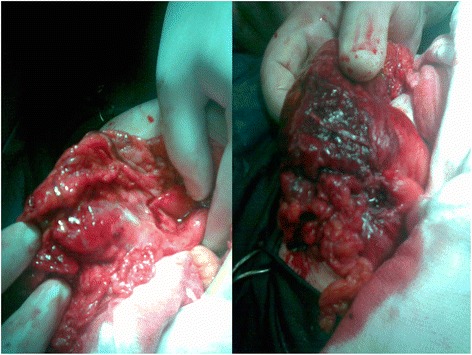
Emergency appendectomy for an incarcerated Amyand’s hernia revealed a malignant rupture.

Our patient underwent surgery, which revealed an incarcerated right inguinal hernia. The hernia sac included her appendix as well as a possibly malignant cecal tumor, which had ruptured in the sac (Figure 2). Immediately, the right groin incision was slightly extended upwards and a right hemicolectomy was performed. Βiopsy was taken and results returned before the hemicolectomy was performed. A histological examination of a biopsy specimen showed adenocarcinoma of the cecum, stage Dukes B.

The postoperative course for our patient was uneventful but for a postoperative lung respiratory infection. She was discharged on the ninth postoperative day.

## Discussion

Amyand’s hernia was first described by Claudius Amyand in 1735, when he performed an appendectomy through a groin incision [1]. Amyand’s hernia is considered rare, with prevalence estimated to be around 1% among all known cases of incarcerated right inguinal hernias, most cases being identified incidentally during surgery [2]. They are more common in men and occur in people of all ages, from premature babies to elderly patients [3,4].

The majority of Amyand’s hernia cases involve right-sided inguinal hernias, most of which present with groin pain. Few cases of left-sided inguinal Amyand’s hernia have been published; those that have were in patients with intestinal malrotation or a mobile cecum [5,6].

Reports in the literature show that an appendix located within an inguinal sac is more frequently inflamed compared to an appendix located normally within the abdominal cavity. An explanation for this finding could be that when an appendix is located within a hernia sac inside the inguinal canal it is more vulnerable to injury and secondary inflammation [7]. In addition, contractions of the abdominal muscles can cause intermittent compression of the appendix, which leads to ischemia of the appendix, infection and severe inflammation [7]. Although localization of the appendix within the inguinal canal does not always lead to appendicitis, this finding is not unusual [8,9].

The major complications of Amyand’s hernia include perforation of the appendix [10], necrotizing fasciitis of the anterior abdominal wall and secondary intestinal perforation. Kueper *et al*. reported that perforation of an Amyand’s hernia can cause a peri-appendicular abscess [11]. This type of hernia can also present with testicular ischemia in newborn babies [12], hyperemia, and hemorrhagic infiltration into the hernia sac [13]. Francko *et al*. published a report of an Amyand’s hernia variant in which the appendix was localized in the inguinal canal, but undetected in the hernia sac [14].

An Amyand’s hernia is therefore a rare condition and is often diagnosed incidentally during hernia surgery. Quite often, the diagnosis is only made in the operating room [15]. It is often connected with rupture and peritonitis and can be a life-threatening condition, with mortality rates ranging between 14% and 30% [9,16].

Losanoff *et al*. proposed a classification system for Amyand’s hernias. When there is no described appendix inflammation, the hernia is classified as type I; type II describes acute appendicitis within the hernia sac; and type III acute appendicitis complicated with peritonitis. Finally, type IV acute appendicitis is accompanied by other diseases [17].

Regarding the treatment of Amyand’s hernias, many authors suggest that prophylactic appendectomy is not necessary when the appendix is found without signs of inflammation [18,19]. By contrast, other authors suggest that all patients have an appendectomy because the appendix could reherniate or, especially in younger patients, cause appendicitis [20]. Prophylactic resection of a left Amyand’s hernia should be performed in many cases because a mobile vermiform appendix to mobile cecum can be reherniated or lead to appendicitis [21].

However, an appendectomy increases the surgical risk and could spread infection to an otherwise clean surgical field, which leads to unnecessary risk for superficial or deep infection [20,21]. In some cases of appendicitis, it has been suggested that surgical repair of the hernia increases wound contamination and fistula formation, and may lead to hernia recurrence [22]. In cases with a non-inflamed appendix, some authors suggest the use of a mesh to treat an Amyand’s hernia [23,24].

There have been few cases reported of a malignancy of the appendix coexisting with an Amyand’s hernia [25,26]. This may be because neoplasms of the appendix are rare and only found in 1% of cases. Most malignancies are carcinoids (80%) [25,26]. It is noteworthy that a number of cases have been reported of mucoceles associated with left colon cancer; in these cases, resection should be performed [26].

## Conclusions

Acute appendicitis is a potential complication of an Amyand’s hernia. In these cases, the appendix appears in the inguinal sac, is inflamed, and the hernia is usually diagnosed incidentally. During surgery for an incarcerated inguinal hernia, we should also keep in mind that malignancy can be involved.

## Consent

Written informed consent was obtained from the patient for publication of this case report and any accompanying images. A copy of the written consent is available for review by the Editor-in-Chief of this journal.
